# Polymers from Bamboo Extracts Produced by Laccase

**DOI:** 10.3390/polym10101141

**Published:** 2018-10-12

**Authors:** Jing Su, Cheng Wang, Jennifer Noro, Artur Cavaco-Paulo, Carla Silva, Jiajia Fu

**Affiliations:** 1 International Joint Research Laboratory for Textile and Fiber Bioprocesses, Jiangnan University, Wuxi 214122, China; jingsu@ceb.uminho.pt (J.S.); 18352537241@126.com (C.W.); artur@deb.uminho.pt (A.C.-P.); 2 Centre of Biological Engineering, University of Minho, Campus de Gualtar, 4710-057 Braga, Portugal; jennifer.noro@ceb.uminho.pt; 3 Jiangsu Sunshine Group Co., Ltd, Jiangyin 214426, China

**Keywords:** bamboo powder, phenolic compound extraction, laccase, oxidation, bamboo tablets

## Abstract

A green methodology for the production of polymers from bamboo powder was investigated. The optimal conditions for the extraction of components from bamboo were defined by incubating the powder in an acetate buffer (pH 5) under boiling for 2 h. Native laccase from *Myceliophthora thermophila* was used afterwards to oxidize the extracts from the final resulting extraction liquid. The reduction of the free OH content after enzymatic oxidation, as well as the ^1^H NMR data, confirmed the efficient polymerization of the extracts. The bamboo powder samples were also subjected to high compression and curing, in the absence and in the presence of laccase, to evaluate the hardness of the tablets formed by enzymatic bonding events. The results revealed a higher hardness when the tablets were produced in the presence of laccase, confirming the role of the catalyst on the precipitation of colloidal lignin and phenolic extractives. Herein we produce new oligomers/polymers by laccase oxidation of the extracts resulting from a clean method boiling. At the same time, the data open up new routes for the exploitation of new lignocellulosic materials by the direct application of the enzyme on the bamboo powder material.

## 1. Introduction

Bamboo, a type of renewable and sustainable natural resource with a powerful regeneration ability, has been investigated in a variety of industrial applications in recent years [1,2]. As a fast-growing forestry plant it requires merely two months growing from a shoot to an adult, and is composed mainly of three polymers including lignin, cellulose, and hemicellulose [3,4]. Hemicellulose and lignin are the most abundant aromatic biopolymers which show an increased importance on the extraction approaches due to their extensive application on biomaterials [5]. Bamboo components, when extracted, can find a panoply of applications that go from antioxidants products to fiberboards, material composites, among others [6,7,8,9]. Moreover, the by-products obtained from bamboo processing can be a cheap source of bioactive compounds, especially antioxidants [10,11]. The development of these new materials might generally involve the addition of chemical adhesives or greener methods like the use of enzymes, namely, oxidoreductases. Laccases, defined as green and effective oxidoreductases, can catalyze an extensive range of substrates such as phenolic compounds and their derivatives [12,13,14,15]. In the recent decades, it has been proved to use laccases in the process of phenolics polymerization under mild conditions, as a potential alternative to the traditional polymerization procedures involving hazardous chemicals and harsh conditions [16,17,18,19,20]. Zerva and Manos investigated the process of bioconversion of phenol monomers using laccase, producing polymers derived from gallic acid and catechol successfully [21]. Liu studied the enzymatic treatment on mechanochemical modified bamboo to extract non-cellulosic matters from finer bamboo fibers in nature, and laccase was used to loosen the compact structure of bamboo fibers [8]. Kumar attempted the procedure of the simultaneous pretreatment and saccharification of bamboo to produce fermentable sugars using laccase [22]. However, few reports on the applications of laccase concerning woody materials could be found, especially related to the laccase polymerization of bamboo powder extracts.

Based on the researches of Higuchi and Wen [23,24], we recently explored the extraction of bamboo bast fiber powders and identified different compounds [25], namely, vanillic acid, 5-hydroxymethylfurfural, gallic acid, guaiacol and vanillin, whose molecules are involved in the lignin biosynthesis of bamboo along with the lignin monomers (LM) of coniferyl alcohol and sinapyl alcohol and some other mono- and oligomers of hemicellulose components of bamboo, as shown in Table 1 [25]. The findings revealed boiling and ultrasound as an efficient and green methodology for the extraction of the hemicellulosic and phenolic compounds from woody bamboo without the addition of any harmful solvents.

In the present work, our goal was to take advantage of bamboo as a cheap source of bioactive compounds like phenolics and test the oxidative ability of laccase to polymerize the extracts obtained from bamboo powder boiling. At the same time, it was our aim to explore the potentiality of this catalyst to be applied directly onto the crude material for the precipitation and bonding of the phenolics and produce novel harder bamboo-based materials. 

## 2. Material and Methods

### 2.1. Material

Commercial food grade bamboo poles originating from Mount Huangshan, Huangshan, China, were used in this study. Folin–Ciocalteu, 2,2′-Azino-bis(3-ethylbenzothiazoline-6-sulfonic acid) (ABTS), Na_2_CO_3_, and acetic acid were purchased from Sigma Aldrich (St. Louis, MO, USA). Laccase (EC 1.10.3.2.) from *Myceliophthora thermophila* was supplied by Novozymes, Copenhagen, Denmark. All other experiment reagents were of analytical grade and used as received, if not otherwise specified.

### 2.2. Extraction of Bamboo Powder 

#### 2.2.1. Bamboo Powder Production

The bamboo poles were cut into small bamboo chips manually for further treatment. The chips were then ground to bamboo powders with the YF-1000 medicine pulverize machine (Yongli, Guangzhou, China). The obtained bamboo powders were then dried to a constant weight in an oven set at 40 °C and stored for further use.

#### 2.2.2. Boiling Extraction of Bamboo Powder in Acetate Buffer

Prior to enzymatic polymerization with laccase, 10 g of bamboo powders in 100 mL of acetate buffer (pH = 5) in a bath ratio of 1:10 were boiled for 2 h and afterwards, the liquid resulting from extraction was recovered and centrifuged (8000 rmp) to completely separate any remaining powder content from the extracted liquid. The remaining bamboo solid was dried to a constant weight in an oven set at 40 °C and stored.

To evaluate the effect of the boiling duration on the amount of extracts obtained, sets of experiments were conducted by boiling for 2 h using the material firstly boiled under the same conditions. In one set of experiments, the liquid of the second extraction was then collected, freeze-dried, and weighed and in the other set the liquid was kept for further treatment with laccase.

### 2.3. Laccase-Assisted Polymerization of Bamboo Extracts

The laccase-assisted polymerization of bamboo extracts was performed using laccase on the liquids resulting from bamboo powder extraction (1st and 2nd boiling). A total of 20 mL of the extraction liquid were incubated with laccase (100 U/mL; specific activity: 4000 U/mL) at 40 °C in a water bath (Grant, OLS Aqua Pro) for 3 h. The experiment control consisted of the incubation of the extracted liquid under the same conditions without the addition of laccase. The polymerization was follow-up by UV/Visible spectroscopy.

#### 2.3.1. Free OH Content by the Folin–Ciocalteu Method

The free OH content was evaluated after laccase oxidation by using the Folin–Ciocalteu method [26]. Herein, 500 µL of Folin solution was added to a 100 µL solution, followed by 6 mL of water, before shaking them for one minute. A total of 2 mL of Na_2_CO_3_ (15% *w*/*v*) and 1.4 mL of water were supplemented afterward, reaching a total volume of 10 mL. After 2 h of reacting adequately at room temperature, the total phenolic compound content of all the samples above was detected at 750 nm. The total content of free OH was assessed by plotting a gallic acid calibration curve (from 1 to 1500 µg/mL). The equation of the gallic acid calibration curve was A = 0.2977c + 0.0368, and the correlation coefficient was r^2^ = 0.9988.

#### 2.3.2. Laccase Activity

The enzymatic activity of laccase was evaluated spectrophotometrically to evaluate its stability during processing. Aliquots of the enzyme solution were taken at different periods of incubation and the activity of laccase was measured against ABTS according to the methodology described by Childs and Bardsley [27]. The enzyme diluted solution was mixed (1:1) with ABTS (5 mM) in an acetate buffer and the increase in absorbance was followed at 420 nm every minute until 10 min of incubation. The spectrophotometer was zeroed with the ABTS_zero_ sample, which contained a mixture of acetate buffer (0.1 M, pH = 5) and ABTS solution. The experiment was performed at 25 °C. The activity in units (U) was defined as the amount of enzyme required to oxidize 1 µmol of ABTS per minute and was calculated by Equation (1) [27]:

(1) $$\;\mathrm{Enzyme}\;\mathrm{activity}\;=\;\frac{\Delta \mathrm{OD}\;*\;V_{total}\;}{\;\varepsilon_{ABTS}*\;d\;*\;V_{enzyme}}\;*\;N\;$$

$V_{total}:$ total volume of test solution; $V_{enzyme}:$ total volume of enzyme solution; $\varepsilon_{ABTS}$: 36,000 L/ (mol* cm); *d*: light pass length (cm); N: enzyme dilute times.

### 2.4. New Materials of Bamboo

#### 2.4.1. Production of Bamboo Powder Tablets

The bamboo tablets were prepared in the raw powder and the powder was boiled for 30 min in a water bath and in an ultrasound bath. Each sample was produced by the preparation of different layers as follows: (i) 0.05 g bamboo + 200 µL acetate buffer + 40 µL laccase + 0.05 g bamboo + pressing and placing in oven (30 min, 40 °C) + tableting for 5 min (pressure: 5000 kg), and the respective control was prepared: (ii) 0.05 g bamboo + 240 µL acetate buffer + 0.05 g bamboo + pressing and placing in oven (30 min, 40 °C) + tableting for 5 min (pressure: 5000 kg). The prepared tablets were then tested for hardness. 

#### 2.4.2. Hardness Testing

The hardness testing of bamboo powder tablets was performed using the TA.HD plus Texture Analyser (Vienna, Austria). Test mode: compression; Pre-Test Speed: 0.50 mm/s; Test speed: 1.00 mm/s; Post-Test Speed: 10.00 mm/s; Target mode: strain (80.0%); Trigger force: 20.0 g; Selected probe: P/25, 25 mm DIA CYLINDER ALUMINIUM. 

## 3. Results and Discussion

### 3.1. Extraction Yield

In a previous work, we defined the optimal conditions for the extraction of hemicelluloses and phenolic compounds from bamboo bast fibers. After testing, 2 h of boiling was set up as the optimal time to extract the maximum amount of compounds from those materials [25]. Taking into account our previous findings, we used the same boiling time to conduct the boiling of the bamboo. The extraction yield was evaluated by weight quantification before and after the boiling processing. From the data obtained (Table 2), one can observe that after the first boiling an extraction yield of 9.19% was achieved while 7.31% was obtained after the second boiling. The two consecutive boilings allowed to achieve a total extraction of 16.50% of the initial material, which is an acceptable working amount considering that no additional solvents are included in the system. One can also verify that with each consecutive boiling, it is possible to extract an additional amount of material, however, with a descending tendency with the number of boilings.

### 3.2. Enzymatic-Assisted Polymerization of the Extracts 

The extraction liquids were oxidized by laccase after extraction and the polymerization was followed spectrophotometrically (λ = 270 nm) and visually as presented in Table 3. The ratio of monomer/enzyme used for the oxidation of the extracts was defined according to a feasible and well-established methodology described by us on several works related with the oxidation of phenolic compounds, and the best operational conditions established were taken into consideration [28,29,30,31]. In fact, we have previously optimized the oxidation of phenolics using laccase in order to achieve a feasible and cost-effective methodology. The overall methodology herein described involved mild reaction conditions, not only at the boiling stage but also on the oxidation step, and might be thus considered a cost-effective process. Considering our previous products’ identification, it is possible to ensure the presence of different phenolic and lignin compounds which are susceptible to be polymerized by laccase, however, since we have not performed their identification herein, it is not possible to identify the major bioactive compounds that were extracted and suffered oxidation by laccase. One can only confirm that polymerization occurred and is detectable by UV/Visible spectroscopy. The data obtained reveal a clear color change from light yellow to dark brown after enzymatic oxidation. This coloration is related to the polymerization promoted by laccase of the different phenolics extracted, previously identified as being vanillic acid, 5-hydroxymethylfurfural, gallic acid, guaiacol, and vanillin [23]. It is noteworthy that a stronger coloration of the samples was visible after the first boiling than after the second one. In the latter case, the amount of extracts is lower, as depicted visually, and a low amount of material is available for polymerization. This is the first evidence to prove that even if one increased the time of boiling, it would not be translated into a significant increase in the amount of material extracted susceptible to be polymerized. 

### 3.3. Free OH Content after Polymerization

**Figure 1:**
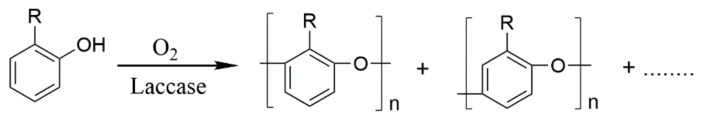
The proposed enzymatic polymerization of phenols assisted by laccase [21].

During the bioconversion of phenolic monomers assisted by laccase, a complex combination of structures can be obtained, as proposed in Figure 1 [21,31]. The studies show that during the polymerization of phenolic monomers, like catechol, the literature reports a typical polymer structure where the catechol units are connected by ether linkages. The reaction occurs by oxygen-carbon bonding at the para-position of the other monomeric unit. This position is more prone to be involved in the reaction binding rather than the *ortho*-position, which involves more stereochemical impediments [21,32,33,34]. Considering this type of reaction, the free OH groups of the oligomers/polymers can be quantified and a decrease of the OH content compared with the monomer (control) is an effective proof of the polymerization [30].

**Figure 2:**
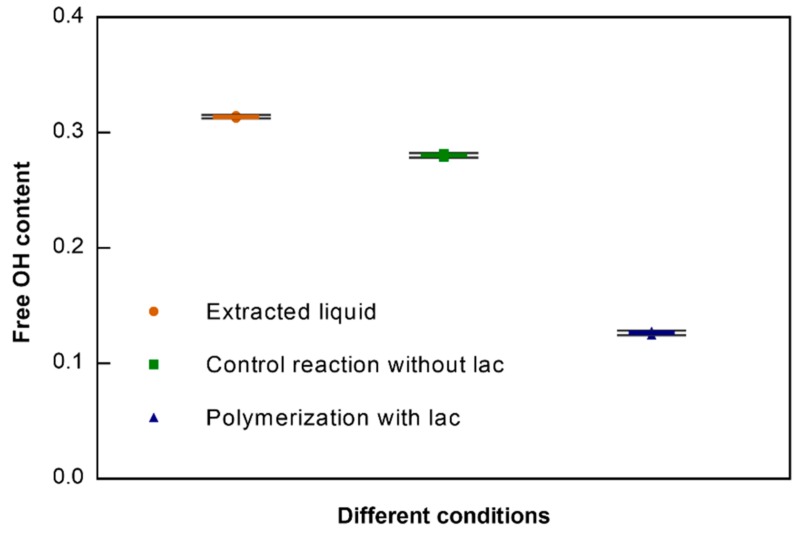
The total content of free OH groups on the extracted liquid and reaction mixtures (data after polymerization of the extracted liquid after the 1st boiling, all the data presented are the mean of 3 repetitions).

For this, the free OH content of the reaction mixtures was evaluated via the Folin–Ciocalteu method [26]. As shown in Figure 2, in the presence of laccase, a significant decrease in the content of the free OH groups can be observed, confirming polymerization. The results are in accordance with the previous UV/Visible data, where the reactions using laccases were responsible to impart higher coloration to the solutions, meaning an effective polymerization, which is confirmed by the lower content of the free OH groups. 

### 3.4. ^1^H NMR Spectroscopy

**Figure 3:**
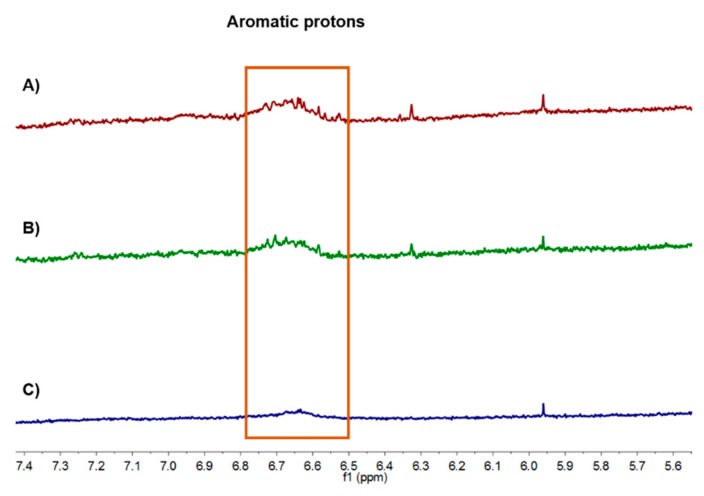
The ^1^H NMR spectra of the bamboo extracted liquid: (**A**) original extracted liquid; (**B**) control reaction without laccase and (**C**) solution after polymerization with laccase (100 U/mL; 40 °C; in a water bath).

After the polymerization of the phenolics extracted from bamboo, the ^1^H NMR spectra were recorded. From the data obtained, it is possible to observe a decrease in the signal intensity of the aromatic protons (δ_H_ 6.7 ppm) (Figure 3C). In the control experiments (Figure 3B) without laccase, the peaks remained the same as in the original liquid of extraction (Figure 3A), confirming the polymerization of the phenolic compounds present in the extraction liquid. 

From the data obtained, it is hard to infer the possible structures obtained after polymerization. The boiling extraction, as mentioned before, gave rise to extractives containing a mixture of compounds which disabled the accurate interpretation of the spectra and the prediction of the polymer structures obtained. The presence of hemicelluloses in the extraction solution might be responsible for the impossibility to accurately acquire the mass spectra of the polymerized solutions by MALDI-TOF (data not shown).

### 3.5. Polymerization Yield 

A simplistic but accurate method was applied to evaluate the polymerization yield after oxidation of the extracts by laccase. The weight difference verified before and after laccase-assisted oxidation gave us an idea of the amount of material formed after the oligomerization/polymerization promoted by laccase in a water bath reactor. One might verify from Table 4 that the polymerization yields were different between the 1st and the 2nd boilings, being higher in the latter. These results might be explained by the oxidation constrains found by laccase in contact with the material firstly extracted. As it is currently reported, the bamboo materials are composed by phenolics, lignin compounds, and hemicelluloses, which are present in a considerable amount in the first boiling extractives, as we previously confirmed [25], and might hinder the laccase oxidative behavior.

### 3.6. Laccase Stability 

**Figure 4:**
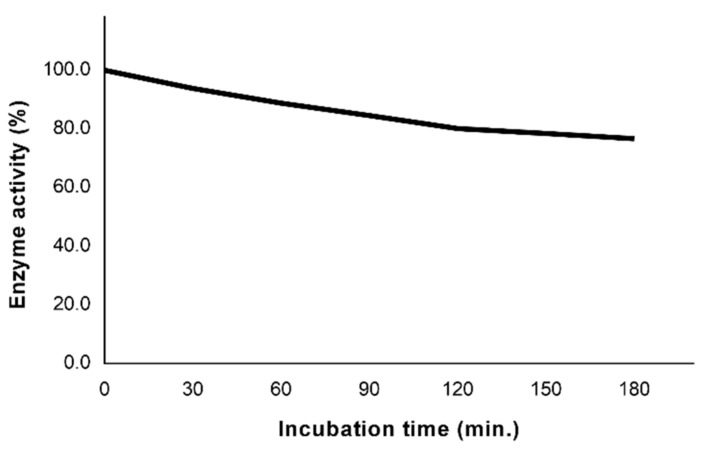
The residual laccase activity after incubation of laccase using a water bath.

Generally, the activity and stability of enzymes are greatly influenced depending on the media used and the time of incubation [35,36]. To predict this, the residual activity of laccase along time was assessed (Figure 4). As observed from Figure 4, the activity of laccase incubated in a water bath showed a slight decrease of the activity after the first hour of incubation (≈90% of the initial activity), losing only 23% of the initial activity after the total time of incubation, 3 h. The data revealed that the catalyst is highly stable in the conditions applied, especially in the first hour of incubation, the period in which the main enzymatic events occur. The data obtained allowed us to infer the possibility of exploring the enzyme recovery after the enzymatic oxidation with the consequent reduction of the costs associated. 

### 3.7. The Hardness of Bamboo Tablets

The concept of the cohesion of the lignin phenolic hydroxyl groups has been established by several authors including Felby et al. [6,37]. The use of lignin-oxidizing enzymes for bonding applications is inspired by the reactivity of phenoxy radicals in the plant cell wall. In vivo, oxidoreductases catalyze the polymerization of lignin through the cross-linking of phenoxy radicals, and a similar type of reaction for the bonding of lignocellulosic materials in vitro can be undertaken [7].

**Figure 5:**
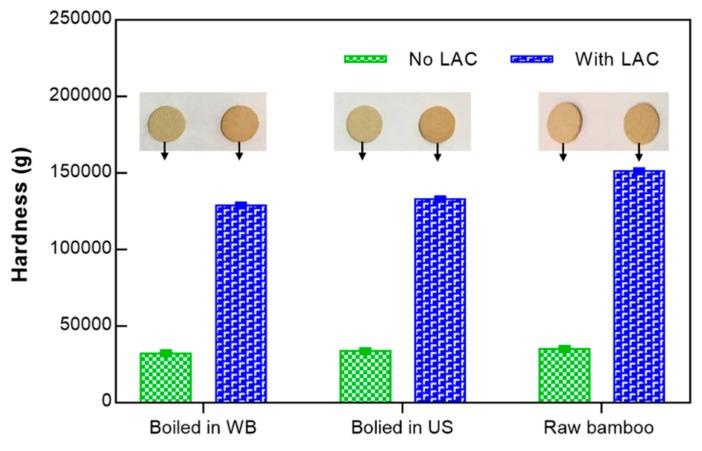
The hardness of the raw bamboo tablets and of the bamboo tablets produced after 30 min of boiling using a water bath and an ultrasonic bath; the tablets were produced by laccase action and heating at 40 °C, under 5000 kg of pressure (the hardness values are the mean of 2 repetitions).

Herein, we used laccase as a catalyst for the bonding of the phenoxy radicals of bamboo to produce a “glued” and compact structure. After production, the tablets were subjected to hardness studies to evaluate the ability of laccase, in conjugation with a high pressure, on bonding and precipitation of colloidal lignin and phenolic extractives. The results presented in Figure 5 reveal a significant increase in the hardness of the bamboo tablets after bonding with laccase, compared with the controls without the enzyme. It is also possible to perceive that a previous boiling of 30 min in a water bath or in an ultrasonic bath did not favor the hardness comparing with the raw bamboo. The extraction of the phenolics to the liquid during boiling decreased the number of phenoxy groups available, therefore hindering the bonding of the lignocellulosic material. 

## 4. Conclusions

In this work, a green methodology to produce polymers from bamboo was developed. The production of new oligomers/polymers was based on a previous boiling powder extraction followed by the enzymatic oxidation of the phenolic extracts by laccase. We confirmed the efficient extraction of the phenolics by boiling without the addition of any solvent and also the ability of laccase to polymerize the extracts. The successive boiling procedures seemed to play a positive role in the amount of materials extracted, which decreases with the number of consecutive boilings performed. The oxidation of the extracts by a feasible and green technology using laccase as a catalyst gave rise to new polymers.

Novel materials from bamboo powder were also attempted. The cohesion and bonding of the phenolics promoted by laccase lead us to produce hard bamboo tablets with a potentiality for a panoply of applications including fiberboards, isolates, and housing materials, among others.

## Figures and Tables

**Table 1:** The products extracted from bamboo powder using boiling and ultrasonic probe treatments and analyzed by LC-ESI-TOF [25].

Compound Extracted	Concentration (mg/mL)
Coniferyl alcohol	2.60
Sinapyl alcohol	2.19
Glucuronic acid	1.38
5-hydroxymethylfurfural	2.01
Gallic acid	0.77
Vanillic acid	0.14
Guaiacol	1.62
D-xylobiose	1.90
Vanillin	1.68
D-cellobiose	0.19
D-cellotriose	0.07

**Table 2:** The weight changes of the bamboo powder after boiling with a buffer (all the data presented results from the mean of 3 independent experiments).

	Original Bamboo Powder (g)	Bamboo Powder After Boiling (g)	Weight Loss of Bamboo Powder After Boiling (g)	Weight of Liquid After Freeze Drying (g)	Extraction Yield (%)	Total Extraction Yield (%)
**1st boiling**	20.00 ± 0.25	18.45 ± 0.12	1.55 ± 0.13	1.84 ± 0.16	9.19 ± 0.67	16.50 ±1.76
**2nd boiling**	18.45 ± 0.12	18.18 ± 0.16	0.27 ± 0.04	1.35 ± 0.21	7.31 ± 1.09	

**Table 3:** The absorbance (λ = 270 nm) after polymerization of the liquid extracts.

Experiment *	After Extraction	Polymerization with Laccase	Control Group without Laccase
Extracted liquid (1st boiling)	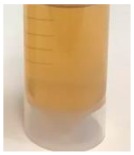	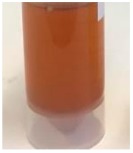	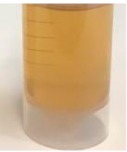
**Absorbance**	**0.468** **± 0.072**	**1.203** **± 0.100**	**0.472** **± 0.035**
Extracted liquid (2nd boiling)	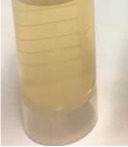	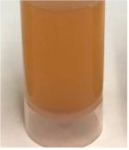	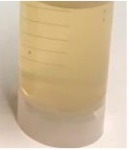
**Absorbance**	**0.403** **± 0.053**	**0.948** **± 0.044**	**0.403** **± 0.028**

* The polymerization of the extracted liquid was conducted at 40 °C in a water bath for 3 h. The experiment control consisted of the incubation of the extracted liquids under the same conditions without laccase (the absorbance values are the results of 3 measurements).

**Table 4:** The polymerization yield after the incubation of the liquid extracts with laccase.

	1st Boiling Extracts	2nd Boiling Extracts
Polymerization yield * (%)	63.0 ± 2.0	73.0 ± 2.0

* Calculated by the difference between the initial weight before oxidation and the weight after oxidation and the centrifugation of the final powder material and removal of the enzyme (the values presented are the mean of 3 repetitions).

## References

[1] Peng H., Hu Z., Yu Z., Zhang J., Liu Y., Wan Y., Ruan R. (2011). Fractionation and thermal characterization of hemicelluloses from bamboo (phyllostachys pubescens mazel) culm. Bioresources.

[2] Li X., Sun C., Zhou B., He Y. (2015). Determination of hemicellulose, cellulose and lignin in moso bamboo by near infrared spectroscopy. Sci. Rep..

[3] Wang H., Zhang L., Zhu D. (2005). An analysis on the characteristics of pyrogenation and carbonization shrinkage of bamboo timber. J. Bamboo Res..

[4] Xu F., Yu J., Tesso T., Dowell F., Wang D. (2013). Qualitative and quantitative analysis of lignocellulosic biomass using infrared techniques: A mini review. Appl. Energy.

[5] Upton B.M., Kasko A.M. (2016). Strategies for the conversion of lignin to high-value polymeric materials: Review and perspective. Chem. Rev..

[6] Felby C., Pedersen L.S., Nielsen B.R. (1997). Enhanced auto adhesion of wood fibers using phenol oxidases. Holzforsch.-Int. J. Biol. Chem. Phys. Technol. Wood.

[7] Felby C., Hassingboe J., Lund M. (2002). Pilot-scale production of fiberboards made by laccase oxidized wood fibers: Board properties and evidence for cross-linking of lignin. Enzyme Microb. Technol..

[8] Liu L., Cheng L., Huang L., Yu J. (2012). Enzymatic treatment of mechanochemical modified natural bamboo fibers. Fibers Polym..

[9] Prosper N.K., Zhang S., Wu H., Yang S., Li S., Sun F., Goodell B. (2018). Enzymatic biocatalysis of bamboo chemical constituents to impart antimold properties. Wood Sci. Technol..

[10] Selvamuthukumaran M., Shi J. (2017). Recent advances in extraction of antioxidants from plant by-products processing industries. Food Qual. Saf..

[11] Zhang Y., Xu W., Wu X., Zhang X., Zhang Y. (2007). Addition of antioxidant from bamboo leaves as an effective way to reduce the formation of acrylamide in fried chicken wings. Food Addit. Contam..

[12] Gonçalves I., Silva C., Cavaco-Paulo A. (2014). Ultrasound enhanced laccase applications. Green Chem..

[13] Giardina P., Faraco V., Pezzella C., Piscitelli A., Vanhulle S., Sannia G. (2010). Laccases: A never-ending story. Cell. Mol. Life Sci..

[14] Jeon J.R., Baldrian P., Murugesan K., Chang Y.S. (2012). Laccase-catalysed oxidations of naturally occurring phenols: From in vivo biosynthetic pathways to green synthetic applications. Microb. Biotechnol..

[15] Arora D.S., Sharma R.K. (2010). Ligninolytic fungal laccases and their biotechnological applications. Appl. Biochem. Biotechnol..

[16] Kim Y.-J., Uyama H., Kobayashi S. (2004). Enzymatic template polymerization of phenol in the presence of water-soluble polymers in an aqueous medium. Polym. J..

[17] Jong-Rok J., Eun-Ju K., Kumarasamy M., Hyo-Keun P., Young-Mo K., Jung-Hee K., Wang-Gi K., Ji-Yeon L., Chang Y.S. (2010). Laccase-catalysed polymeric dye synthesis from plant-derived phenols for potential application in hair dyeing: Enzymatic colourations driven by homo- or hetero-polymer synthesis. Microb. Biotechnol..

[18] Kim S., Silva C., Evtuguin D.V., Gamelas J.A., Cavaco-Paulo A. (2011). Polyoxometalate/laccase-mediated oxidative polymerization of catechol for textile dyeing. Appl. Microbiol. Biotechnol..

[19] Desentis-Mendoza R.M., Hernandez-Sanchez H., Moreno A., Rojas D.C.E., Chel-Guerrero L., Tamariz J., Jaramillo-Flores M.E. (2006). Enzymatic polymerization of phenolic compounds using laccase and tyrosinase from ustilago maydis. Biomacromolecules.

[20] Su J., Fu J., Wang Q., Silva C., Cavaco-Paulo A. (2017). Laccase: A green catalyst for the biosynthesis of poly-phenols. Crit. Rev. Biotechnol..

[21] Zerva A., Manos N., Vouyiouka S., Christakopoulos P., Topakas E. (2016). Bioconversion of biomass-derived phenols catalyzed by myceliophthora thermophila laccase. Molecules.

[22] Kumar S., Gujjala L.K.S., Banerjee R. (2017). Simultaneous pretreatment and saccharification of bamboo for biobutanol production. Ind. Crops Prod..

[23] Higuchi T. (1969). Bamboo lignin and its biosynthesis. Wood Res..

[24] Wen J.L., Xiao L.P., Sun Y.C., Sun S.N., Xu F., Sun R.C., Zhang X.L. (2011). Comparative study of alkali-soluble hemicelluloses isolated from bamboo (bambusa rigida). Carbohydr. Res..

[25] Wang C., Tallian C., Su J., Vielnascher R., Silva C., Cavaco-Paulo A., Guebitz G.M., Fu J. (2018). Ultrasound-assisted extraction of hemicellulose and phenolic compounds from bamboo bast fiber powder. PLoS ONE.

[26] Stankovikj F., Mcdonald A.G., Helms G.L., Olarte M.V., Garciaperez M. (2017). Characterization of woody biomass pyrolysis oils’ water soluble fraction. Energy Fuels.

[27] Childs R.E., Bardsley W.G. (1975). The steady-state kinetics of peroxidase with 2,2′-azino-di-(3-ethyl-benzthiazoline-6-sulphonic acid) as chromogen. Biochem. J..

[28] Su J., Noro J., Fu J., Wang Q., Silva C., Cavaco-Paulo A. (2018). Enzymatic polymerization of catechol under high-pressure homogenization for the green coloration of textiles. J. Clean. Prod..

[29] Su J., Noro J., Fu J., Wang Q., Silva C., Cavaco-Paulo A. (2018). Exploring pegylated and immobilized laccases for catechol polymerization. AMB Express.

[30] Su J., Noro J., Loureiro A., Martins M., Azoia N.G., Fu J., Wang Q., Silva C., Cavaco-Paulo A. (2017). Pegylation greatly enhances laccase polymerase activity. ChemCatChem.

[31] Su J., Castro T.G., Noro J., Fu J., Wang Q., Silva C., Cavaco-Paulo A. (2018). The effect of high-energy environments on the structure of laccase-polymerized poly(catechol). Ultrason. Sonochem..

[32] Aktaş N., Şahiner N., Kantoğlu Ö., Salih B., Tanyolaç A. (2003). Biosynthesis and characterization of laccase catalyzed poly(catechol). J. Polym. Environ..

[33] Sun X., Bai R., Zhang Y., Wang Q., Fan X., Yuan J., Cui L., Wang P. (2013). Laccase-catalyzed oxidative polymerization of phenolic compounds. Appl. Biochem. Biotechnol..

[34] Aktas N., Tanyolac A. (2003). Kinetics of laccase-catalyzed oxidative polymerization of catechol. J. Mol. Catal. B Enzym..

[35] Rokhina E.V., Lens P., Virkutyte J. (2009). Low-frequency ultrasound in biotechnology: State of the art. Trends Biotechnol..

[36] Delgado-Povedano M.M., Luque de Castro M.D. (2015). A review on enzyme and ultrasound: A controversial but fruitful relationship. Anal. Chim. Acta.

[37] Gouveia S., Otero L., Fernández-Costas C., Filgueira D., Sanromán Á., Moldes D. (2018). Green binder based on enzymatically polymerized eucalypt kraft lignin for fiberboard manufacturing: A preliminary study. Polymers.

